# Facial Cutaneous Metastasis in Renal Cell Carcinoma

**DOI:** 10.7759/cureus.12093

**Published:** 2020-12-15

**Authors:** Deyson Lorenzo-Rios, Estefania Cruzval-O'Reilly, Jose Rabelo-Cartagena

**Affiliations:** 1 Dermatology, University of Puerto Rico, San Juan, PRI; 2 Dermatology, Universidad Central del Caribe School of Medicine, Bayamón, PRI; 3 Dermatology, Veterans Affairs Caribbean Health System, San Juan, PRI

**Keywords:** renal cell carcinoma, cutaneous metastasis, neoplasm metastasis, skin neoplasm

## Abstract

Renal cell carcinoma (RCC) is a common genitourinary malignancy of increasing incidence and significant mortality rate. Skin metastases of RCC are considered a rare phenomenon of unfavorable outcomes. We present a 75-year-old male patient who developed a rapidly evolving lesion on his left cheek four years after undergoing a right radical nephrectomy for non-metastatic RCC. Immunohistochemistry of the skin lesion was diagnostic for cutaneous metastasis of renal clear cell carcinoma, which eventually led to the detection of internal malignancy recurrence by positron emission tomography. A new facial skin lesion may unmask the underlying recurrence of RCC.

## Introduction

Renal cell carcinoma (RCC) is a genitourinary malignancy of male predominance with an annual incidence of over 73,000 cases in the United States and a five-year relative survival rate of 75% [1,2]. In recent decades, improvements in imaging have allowed for earlier recognition of disease. However, only 65% of patients with RCC present with localized disease, while 16% present with metastatic disease [1,2]. Metastasis to the skin from RCC is considered a rare phenomenon, as the most common sites of metastasis are lung, bone, liver, lymph nodes, and brain [3]. In this article, we present a male with facial cutaneous metastasis from RCC four years after radical nephrectomy. 

## Case presentation

**Figure 1:**
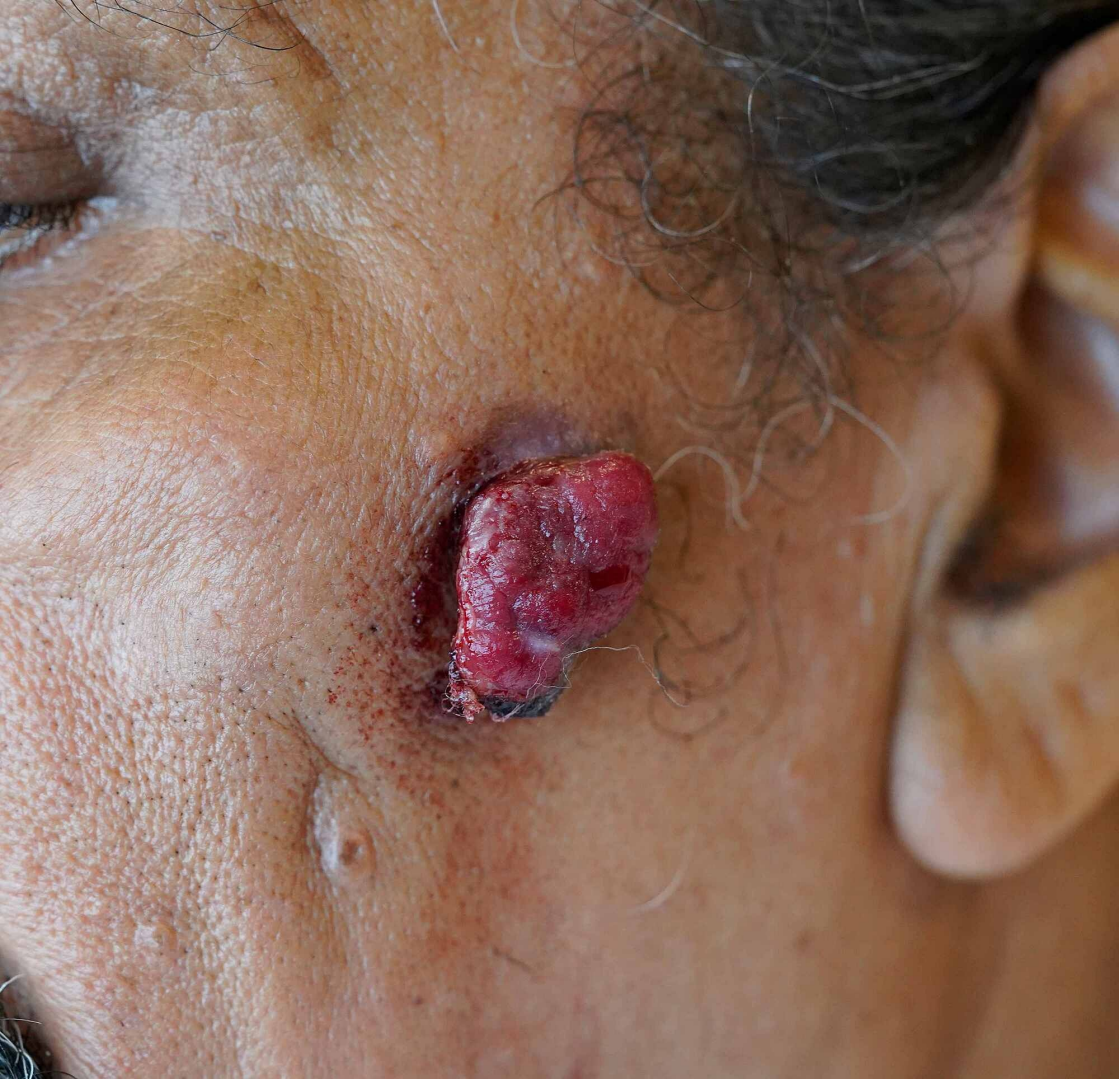
A 1.3 cm × 0.6 cm exophytic tumor with friable consistency on a violaceous base on the patient’s left cheek.

**Figure 2:**
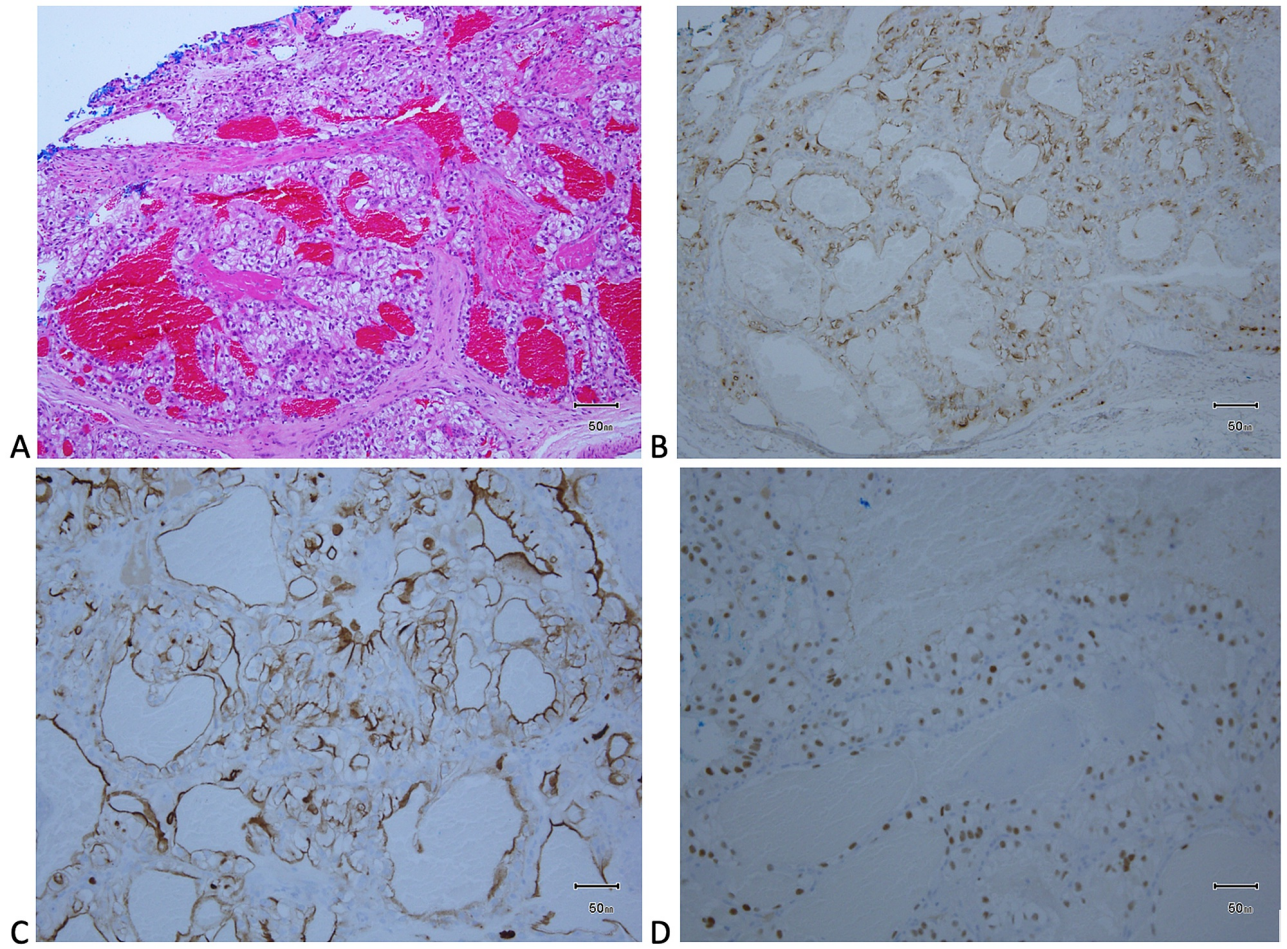
H&E staining showing a non-encapsulated neoplasm composed of clear cells infiltrating into the dermis (A). Immunohistochemistry was positive for RCC-Marker (B), CD-10 (C), and PAX-8 (D). RCC: renal cell carcinoma, H&E: hematoxylin and eosin.

A 75-year-old Hispanic man with a history of T1bN0M0 clear cell RCC, managed with right radical nephrectomy four years prior, presented with a facial skin lesion of four months of evolution. The lesion started as a small crusted papule with occasional bleeding that rapidly grew into a tender tumor. Annual surveillance of his RCC, which included a CT scan, a physical examination, and routine labs, was uninterrupted and consistently negative for metastatic disease. The patient denied fatigue, fever, weight loss, shortness of breath, flank, or abdominal pain. Physical examination revealed a 1.3 cm × 0.6 cm exophytic, pedunculated tumor with friable consistency on a violaceous base on the left cheek (Figure 1). No lymphadenopathy, abdominal masses, or buccal lesions were found. Laboratories revealed normocytic normochromic anemia, stage 3 chronic kidney disease, and elevated alkaline phosphatase. Calcium levels and urinalysis were within normal limits. A shave biopsy with debulking of the lesion was performed. Microscopic analysis of the specimen showed individual cells with abundant clear cell cytoplasm with small nuclei of bland appearance. An increased number of vascular channels were also seen, correlating with frequent bleeding and friable appearance. Histopathology was consistent with RCC of clear cell type, extending into the dermis (Figure 2A). The differential diagnoses considered included pyogenic granuloma, basal cell carcinoma, angioma, keratoacanthoma, and cutaneous metastasis. Pyogenic granuloma was considered given the rapid growth, location, and friable appearance of the lesion. However, the patient’s advanced age and medical history together with the evolution of the skin lesion suggested a possible cutaneous metastasis despite the unusual distal location and absence of constitutional symptoms. Tissue staining was positive for RCC-marker, CD10, and PAX8 immunoperoxidase markers, supporting the diagnosis of cutaneous metastasis of RCC (Figures 2B-2D).

 

**Figure 3:**
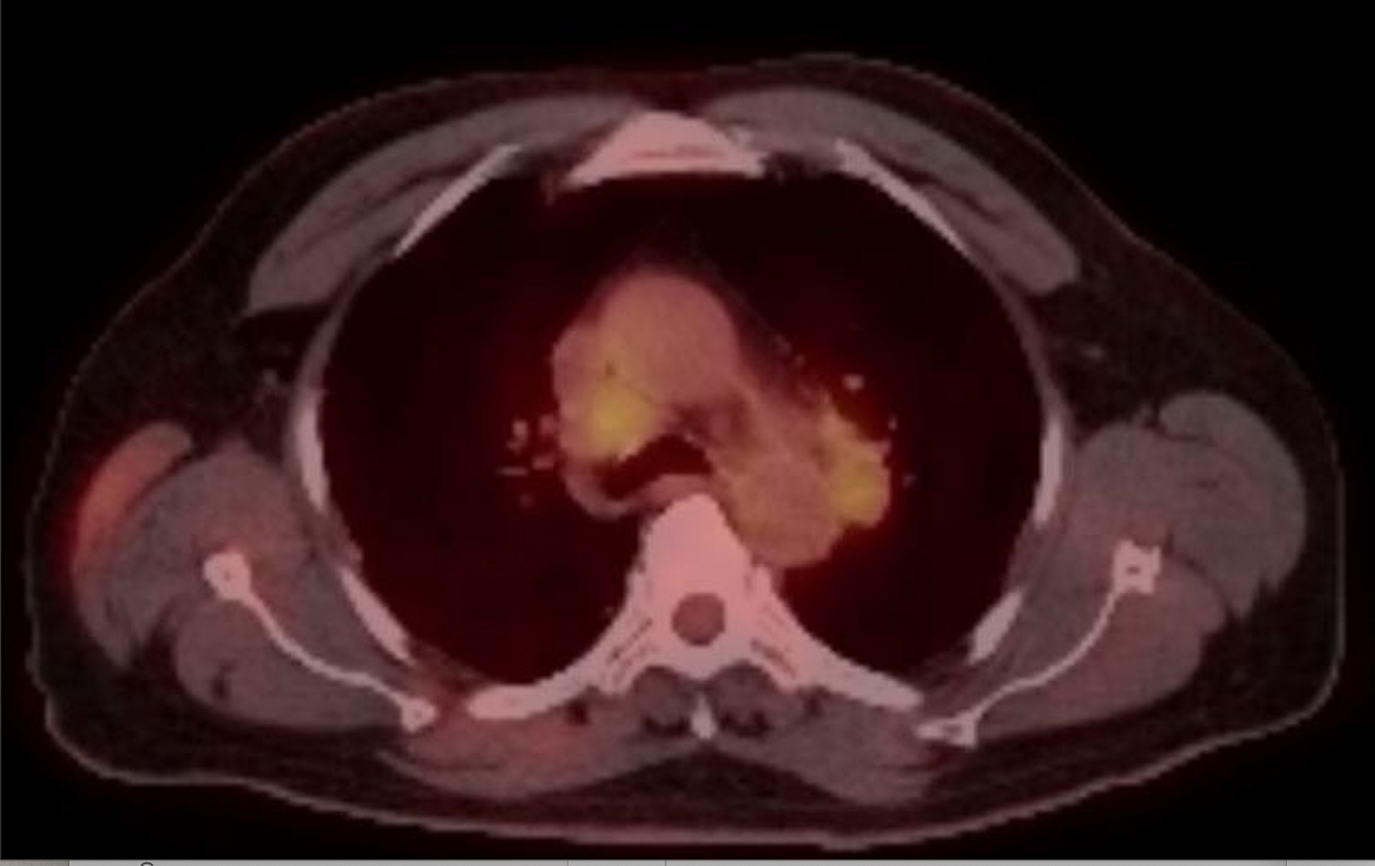
PET/CT showing hypermetabolic mediastinal lymph nodes in the precarinal and left prevascular spaces. PET/CT: positron emission computerized tomographic scanning.

An excisional surgery was performed by a plastic surgeon and specimen margins were free of neoplasm. A positron emission computerized tomographic scanning (PET/CT) revealed extensive bilateral lung nodules, mediastinal lymphadenopathy, and a left distal femoral lesion, all with metabolic activity concerning metastatic disease (Figure 3). The patient was started on immunotherapy with the combination of nivolumab and ipilimumab and completed four cycles of treatment every 21 days. A repeat PET/CT scan revealed diminished metabolic activity and number of lytic lesions, and a reduction of size and number of lung nodules, suggesting a response to treatment. Nine months after being diagnosed with metastatic disease, the patient remains on maintenance treatment with nivolumab, which he is tolerating well.

## Discussion

Approximately 25% of patients diagnosed with RCC develop metastatic disease [3]. In some cases, skin lesions rather than the classic symptoms of hematuria, back pain, and a palpable mass have been the chief complaint of patients with undiagnosed RCC [4]. The reported incidence of cutaneous metastases from RCC among patients diagnosed by 2017 was 3.3% with a mean survival of 10.9 months [5]. Cutaneous metastases of RCC are commonly found on the head, chest, and abdomen [5]. Other unusual sites reported include chin [6,7], preauricular area [8], tongue, and fingers [9,10]. Cutaneous metastases from RCC tend to present as a single, rapidly growing, reddish-blue lesion. Patients with a history of RCC are more likely to develop cutaneous metastasis within six months to five years from the time of initial diagnosis and after radical nephrectomy [11]. The postulated mechanism of RCC responsible for cutaneous metastasis to the head and neck is by lymphohematogenous extension [8]. Because patients presenting with cutaneous metastasis are likely to have other concomitant sites of metastasis, skin findings are often considered a sign of end-stage RCC and are associated with a poor prognosis, as patients with RCC and distant metastasis have a five-year survival rate of 12% [2,5].

Skin metastases from RCC vary in consistency, color, size, and texture, clinically resembling sebaceous cysts, angiomas, adnexal tumors, pyogenic granulomas, among others [7,11]. When multiple, they have been found to mimic Kaposi’s sarcoma as well [12]. Clear cells are a highly nonspecific histologic finding as several other malignancies, such as those of skin appendages, urinary tract, endometrium, glandular, and soft tissues, have similar cellularity and may develop distant metastases [13,14]. Clear cell carcinoma subtype accounts for 82% of the cases of cutaneous metastases from RCC, and its molecular characterization has been conveniently and extensively described [5,15].

Besides the cellular description, immunohistochemical markers provided certainty to the diagnosis, revealing an internal malignancy rather than a primary skin neoplasm. PAX8 is a transcription factor found in the kidneys and other internal organs, making it useful in the identification of metastatic tissue [16]. Moreover, CD-10 is a cell surface protein expressed by kidney neoplasms that are present in up to 94% of the clear cell RCC cases [17]. Although PAX8 and CD10 are not exclusive markers for RCC, the monoclonal antibody against the brush border of proximal tubular cells, or RCC-marker, is a more specific marker for carcinoma of renal origin which can be included in the immunohistochemistry, narrowing the differential to renal cell carcinoma [16,17].

Initial workup for cutaneous metastasis should include the assessment of a possible internal primary malignancy that may warrant targeted therapy. As evidenced on the PET/CT scan, our patient was found with a subclinical metastatic disease that correlated with his facial skin lesion. Internal lesions were considered a recurrence of RCC despite radical nephrectomy performed years prior. No evidence of residual disease in the right renal fossa nor metachronous malignancy of the remaining contralateral kidney was found on the PET/CT scan. 

Surgical removal of the skin lesion is recommended not only for cosmesis, but also when there are concerning tumor features such as rapid growth, local extension, and bleeding. Selected cases of cutaneous metastasis of RCC have been considered to undergo radiotherapy [18]. Adjuvant therapy for solitary cutaneous metastases of RCC has not been described so far. Current guidelines recommend nivolumab combined with ipilimumab for patients with advanced RCC without prior exposure to systemic therapy. Treatment with nivolumab/ipilimumab has shown to improve overall survival and progression-free survival compared with sunitinib [19]. Patients with metastatic RCC involving atypical sites, such as a digit and the tongue, have shown variable responses to adjuvant chemotherapy [9,10]. In such cases, a palliative approach has been considered [18]. 

## Conclusions

Although rare, clinicians should be aware of a possible cutaneous metastasis from RCC in patients who present with a rapidly growing, irregular skin lesion. A full skin examination performed during prolonged surveillance may be beneficial in RCC. A comprehensive examination, immunohistochemical analysis, and prompt diagnosis are essential, as this will lead to appropriate treatment and potentially, more favorable outcomes.
